# Co-Clinical Evaluation of Synthetic Vitamin D and Chemotherapy in Pancreatic Cancer by Integrated Imaging and Molecular Analyses

**DOI:** 10.1158/2767-9764.CRC-26-0443

**Published:** 2026-08-25

**Authors:** Mamta Gupta, Hoon Choi, Thomas Karasic, Emma E. Furth, Sydney Shaffer, Sarah Englander, Stephen Pickup, Cynthia Clendenin, Fang Liu, Quy Cao, Hee Kwon Song, Yong Fan, Jeffrey Duda, James C. Gee, Mark Rosen, Peter J. O’Dwyer, Rong Zhou

**Affiliations:** 1 Department of Radiology, University of Pennsylvania, Philadelphia, Pennsylvania.; 2 Abramson Cancer Center, University of Pennsylvania, Philadelphia, Pennsylvania.; 3 Pancreatic Cancer Research Center, University of Pennsylvania, Philadelphia, Pennsylvania.; 4 Department of Pathology, University of Pennsylvania, Philadelphia, Pennsylvania.; 5 Center for Clinical Epidemiology and Biostatistics, University of Pennsylvania, Philadelphia, Pennsylvania.

## Abstract

**Purpose:**

A pilot clinical trial (NCT03519308) was conducted to evaluate perioperative chemotherapy [nab-paclitaxel, gemcitabine, and cisplatin (NGC)] plus nivolumab, with or without synthetic vitamin D (paricalcitol) in resectable pancreatic ductal adenocarcinoma (PDAC). Preclinical studies were designed with statistical power to match the experimental regimen and compare NGC only with NGC plus synthetic vitamin D [calcipotriol (Cal)] or losartan (Losa; an antihypertensive medication).

**Materials and Methods:**

Molecular subtyping was applied to enroll patients with epithelial subtype PDAC. Genetically engineered PDAC models (*Kras*^*G12D*^*;Trp53*^*R172H*^*;Pdx1-Cre* mice, *N* = 140) were randomized to NGC only and combined therapies. Multiparametric MRI and molecular profiling were integrated to assess treatment-induced effects on stroma, including capillary perfusion and density, matrix collagen content, fibroblast activation protein (FAP) levels and transcriptome changes.

**Results:**

The clinical trial had limited enrollment (*N* = 3) but confirmed the safety and feasibility of weekly paricalcitol infusion in combination with multiagent chemotherapy. NGC chemotherapy had a profound impact on stroma, inducing a remarkable reduction in FAP levels accompanied by a significant increase in matrix collagen content; chemotherapy also activated epithelial–mesenchymal transition (EMT) and significantly reduced blood perfusion and capillary density, resulting in features of chemoresistance. Combining NGC with stromal-targeting agents—Cal or Losa—diminished EMT and partially restored perfusion through distinct mechanisms: Cal activates the vitamin D receptor to reprise a quiescent stroma, whereas Losa suppresses TGFβ to increase lymphocyte infiltration.

**Conclusions:**

These findings support further investigations of stromal-directed combination strategies to overcome chemotherapy-induced resistance of PDAC. Dynamic contrast-enhanced MRI may provide a valuable platform to monitor stromal adaptations to therapy *in vivo*.

**Significance:**

Results from co-clinical trials integrating MRI and molecular profiling provide a translational rationale for investigating stromal-directed combination strategies in PDAC. These approaches may help preserve chemosensitivity, counteract chemotherapy-induced stromal and vascular remodeling, and overcome adaptive resistance mechanisms that limit durable treatment response.

## Introduction

Pancreatic ductal adenocarcinoma (PDAC) is characterized by a dense desmoplastic stroma composed of microvasculature, extracellular matrix (ECM), and diverse stromal cells, including pancreatic stellate cells, cancer-associated fibroblasts (CAF), and immune cells (1). Multiagent chemotherapy, such as nab-paclitaxel plus gemcitabine or a combination of folinic acid (leucovorin/calcium folinate), fluorouracil, irinotecan, and oxaliplatin (FOLFIRINOX), has improved survival and become the standard treatment for patients with locally advanced or metastatic PDAC (2–5). Nonetheless, outcomes remain suboptimal because of intrinsic and acquired chemoresistance. The dense stroma contributes to this resistant phenotype by impairing drug delivery, altering tumor perfusion, and supporting cancer cell survival (6–8). Chemotherapy sensitivity is also influenced by tumor molecular subtype: Epithelial/classical tumors tend to respond better, whereas basal or mesenchymal tumors are generally more resistant (9–13).

Early efforts to improve therapeutic delivery by depleting specific stromal components, including hyaluronan (7, 14, 15), or by targeting fibroblast signaling through hedgehog pathway inhibition (16, 17), failed in clinical trials despite encouraging preclinical or early-phase findings. These results suggest that complete stromal depletion may be suboptimal or even counterproductive and support an alternative strategy of stromal remodeling or normalization. One promising approach is the activation of vitamin D receptors (VDR) in pancreatic stellate cells. Synthetic vitamin D analogs, such as paricalcitol and calcipotriol (Cal), have been shown in murine PDAC models to reverse activated stellate cells toward a more quiescent phenotype, remodel the desmoplastic stroma, and enhance gemcitabine delivery (18). This strategy is conceptually distinct from stromal depletion because it aims to remodel the tumor microenvironment rather than simply remove stromal elements.

**Figure 1.**
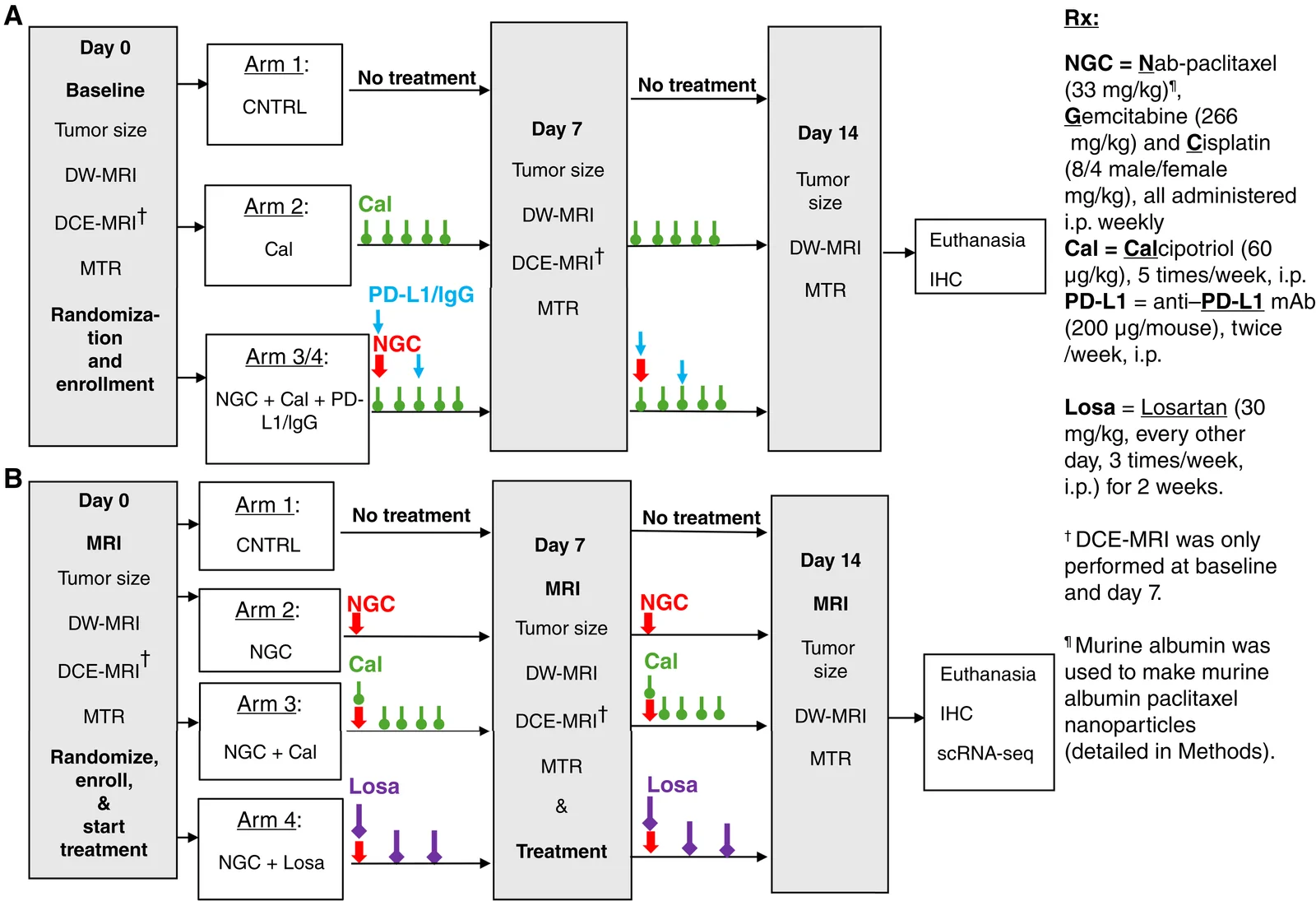
Design of preclinical trials in the KPC model. **A,** The “Matched trial” aimed at testing the matched experimental treatment in arm A of the clinical trial and the effect of synthetic vitamin D–only treatment. **B,** The “chemo–stromal trial” aimed at comparing multiagent chemotherapy (NGC) and its combination with synthetic vitamin D and Losa, respectively. Both male and female mice with MRI-estimated tumor sizes in the range of 104 ± 40 mm^3^ were enrolled on day 0 and randomized into treatment arms. Tumor size was again measured on days 7 and 14 by MRI. DW-MRI and MTR protocols were applied to a subset of enrolled mice on days 0, 7, and 14 due to scanner availability. DCE-MRI was conducted on days 0 and 7 due to the technical challenge of multiple tail vein catheterizations in the same mouse. scRNA-seq, single-cell RNA-seq.

Based on this rationale, we conducted a perioperative pilot trial (NCT03519308) testing nab-paclitaxel, gemcitabine, and cisplatin (NGC) plus nivolumab with or without paricalcitol, an FDA-approved synthetic vitamin D analog, in patients with epithelial-subtype PDAC (Supplementary Fig. S1). Tumor biopsy and subtyping were required to identify epithelial versus basal PDAC because VDR agonism was reported to increase metastatic potential in basal PDAC models in a prior animal study (19). In parallel, we used a genetically engineered *Kras*^*G12D*^*;Trp53*^*R172H*^*;Pdx1-Cre* (KPC) mouse model, which recapitulates key features of human PDAC, including dense stroma (20), to evaluate the matched experimental regimen and Cal monotherapy in a preclinical “matched trial” (Fig. 1A). We also tested NGC alone versus NGC combined with Cal or Losa in a separate “chemo–stromal” trial (Fig. 1B), allowing comparison of two stromal-modulating strategies.

We tested the utility of clinically ready MRI protocols for assessing changes in the tumor microenvironment over time in response to chemotherapy and/or stromal-directed treatments. MRI protocols were implemented in both clinical and preclinical studies, including tumor size measurement, diffusion-weighted MRI (DW-MRI), and dynamic contrast-enhanced MRI (DCE-MRI). Imaging studies were complemented by IHC for tumor cellularity, microvascular density (MVD), matrix collagen, and fibroblast activation protein (FAP) expression. The apparent diffusion coefficient (ADC) derived from DW-MRI provides a noninvasive marker of ECM level and tumor cellularity (21). DCE-MRI enables serial assessment of microvascular perfusion and permeability, parameters relevant to drug delivery (22, 23), whereas MVD can only be assessed from surgical or postmortem tissue (24). Because magnetization transfer ratio (MTR) imaging is sensitive to tissue fibrosis (25), we tested whether it could detect matrix collagen changes in KPC tumors. Finally, we performed a preliminary single-cell RNA sequencing (RNA-seq) study to explore whether treatment induced molecular subtype shifts and to generate hypotheses for future investigation.

## Materials and Methods

### Clinical studies

NCT03519308 was an Institutional Review Board (IRB)–approved randomized pilot perioperative study testing whether paricalcitol could be safely combined with NGC plus nivolumab in resectable PDAC (Supplementary Fig. S1). Written informed consent was obtained from all individual participants prior to their enrollment in this study, which was conducted in accordance with ethical guidelines. Tumor subtype was determined from endoscopic core biopsy, and only patients with epithelial-subtype PDAC were eligible. Although subtype testing was pending, patients received one cycle of chemotherapy. Eligible patients were then enrolled in arm A or arm B. Research MRI was performed at baseline and after the completion of neoadjuvant treatment before surgical resection. Patients consented separately for the research MRI, performed on a 1.5T Siemens Avanto system with T2-weighted, DW-MRI, and DCE-MRI. Tumor size was estimated on T2-weighted images using RECIST-based long-axis measurements. ADC was calculated by fitting diffusion-weighted signal intensities to a single-exponential decay model (23). DCE-MRI was acquired using a three-dimensional stack-of-stars radial sequence after gadolinium contrast injection (26). Tumor T1, K^trans^, and V_e_ were derived from radial acquisitions and kinetic modeling using Tofts and shutter speed models (26). Surgically resected tumor specimens were stained for hematoxylin and eosin (H&E; for cell density), Sirius red (matrix collagen), and CD31 (MVD).

Additional PDAC surgical specimens were obtained for FAP staining from the biobank (IRB Protocol# 853345) from patients who underwent neoadjuvant chemotherapy (NAC; *n* = 10) and untreated (*n* = 10) before surgery.

### Preclinical *in vivo* studies

All animal studies were approved by the University of Pennsylvania Institutional Animal Care and Use Committee. KPC mice were screened by palpation and ultrasound, and MRI was used to confirm tumor size before enrollment. Mice with MRI-estimated tumor volumes of 104 ± 40 mm^3^ were randomized into two preclinical trial designs: The first trial tested the matched experimental regimen in clinical trial arm A (NGC plus anti–PD-L1 antibody and Cal); it also evaluated Cal monotherapy (Fig. 1A). The second trial compared NGC alone with NGC plus Cal or Losa (Fig. 1B). Both male and female mice were used; treatments were given intraperitoneally, and trials lasted 2 weeks. As detailed in Supplementary Methods, a murine albumin formulation of nab-paclitaxel was prepared to avoid immune reactions to human albumin.

Preclinical MRI was performed on a 9.4T Bruker system under isoflurane anesthesia with physiologic monitoring and temperature control, as described earlier (27). T2-weighted MRI was used for tumor volume measurement. DW-MRI, DCE-MRI, and MTR imaging were applied to assess tumor cellularity, vascular function, and tissue macromolecular/fibrotic features. DCE-MRI was performed on days 0 and 7, whereas T2-weighted MRI, DW-MRI, and MTR imaging were performed on days 0, 7, and 14 when available. Tumor regions of interest were manually drawn on T2-weighted images and propagated to quantitative maps. Tumor volume was calculated from summed tumor areas across slices. Pixelwise ADC, kurtosis index, K^trans^, V_e_, and MTR maps were generated, and median tumor values were used for group-level analysis.

### *Ex vivo* studies

Formalin-fixed, paraffin-embedded tumor sections from patients and KPC mice were stained with H&E, CD31, Sirius red, or FAP, as appropriate. Sections were scanned at 40× magnification and analyzed by a gastrointestinal pathologist (E.E. Furth) blinded to treatment information. Whole-section QuPath analysis was used to quantify tumor cellularity, MVD, matrix collagen content, and FAP staining while excluding nontumor tissue. For exploratory single-cell RNA-seq, viable cells from KPC tumors were processed using the 10x Genomics Chromium Single Cell 3′ platform. Data were aligned to the mouse transcriptome using Cell Ranger and analyzed using Seurat (RRID:SCR_007322), SCTransform, SingleR/celldex, and Cirrocumulus. Cell type assignments and epithelial, basal/mesenchymal, and CAF-associated gene programs were interpreted using published marker sets.

### Statistical analysis

Data are reported as mean ± SD unless otherwise specified. Statistical analyses were performed using GraphPad Prism (RRID:SCR_002798) or R. Normality of the data was assessed by Shapiro–Wilk testing. Normally distributed data were compared using a two-tailed *t* test, whereas non-normally distributed data were compared using Mann–Whitney tests. Logistic regression was used to estimate the likelihood of tumor regression for NGC compared with NGC plus Cal or Losa. Sample sizes are given in figure legends. Statistical significance was defined as alpha = 0.05.

Additional details of co-clinical studies, including MRI acquisition parameters, image analysis, IHC, single-cell RNA-seq, and statistical methods, are provided in the Supplementary Methods.

## Results

### Tumor growth and ADC response

Nine patients were recruited for NCT03519308, with demographic data compiled in Supplementary Table S1: Three were enrolled (one in arm A and two in arm B), and six were excluded because of insufficient biopsy tissue for subtyping (*n* = 4) or basal subtype cancer (*n* = 2). The trial was paused and then prematurely terminated because of the COVID-19 pandemic’s impact. The representativeness of study participants is provided in Supplementary Table S2. Despite limited accrual, the study confirmed the safety and feasibility of combining synthetic vitamin D infusion with multiagent chemotherapy and an immune checkpoint inhibitor in the perioperative setting. In the sections below, the exploratory results due to limited patient cases are introduced first, followed by statistically powered data from KPC mice.

As shown in Supplementary Fig. S2A, after neoadjuvant treatment, the tumor long-axis dimension decreased in patient #7 from 1.5 to 0.7 cm and in patient #3 from 3.2 to 2 cm, consistent with partial response by RECIST criteria. Patient #4 was not evaluable because the lesion appeared as a slightly thickened pancreas head on both baseline and posttreatment MRI, whereas H&E staining of the resected specimen identified a small residual tumor.

**Figure 2.**
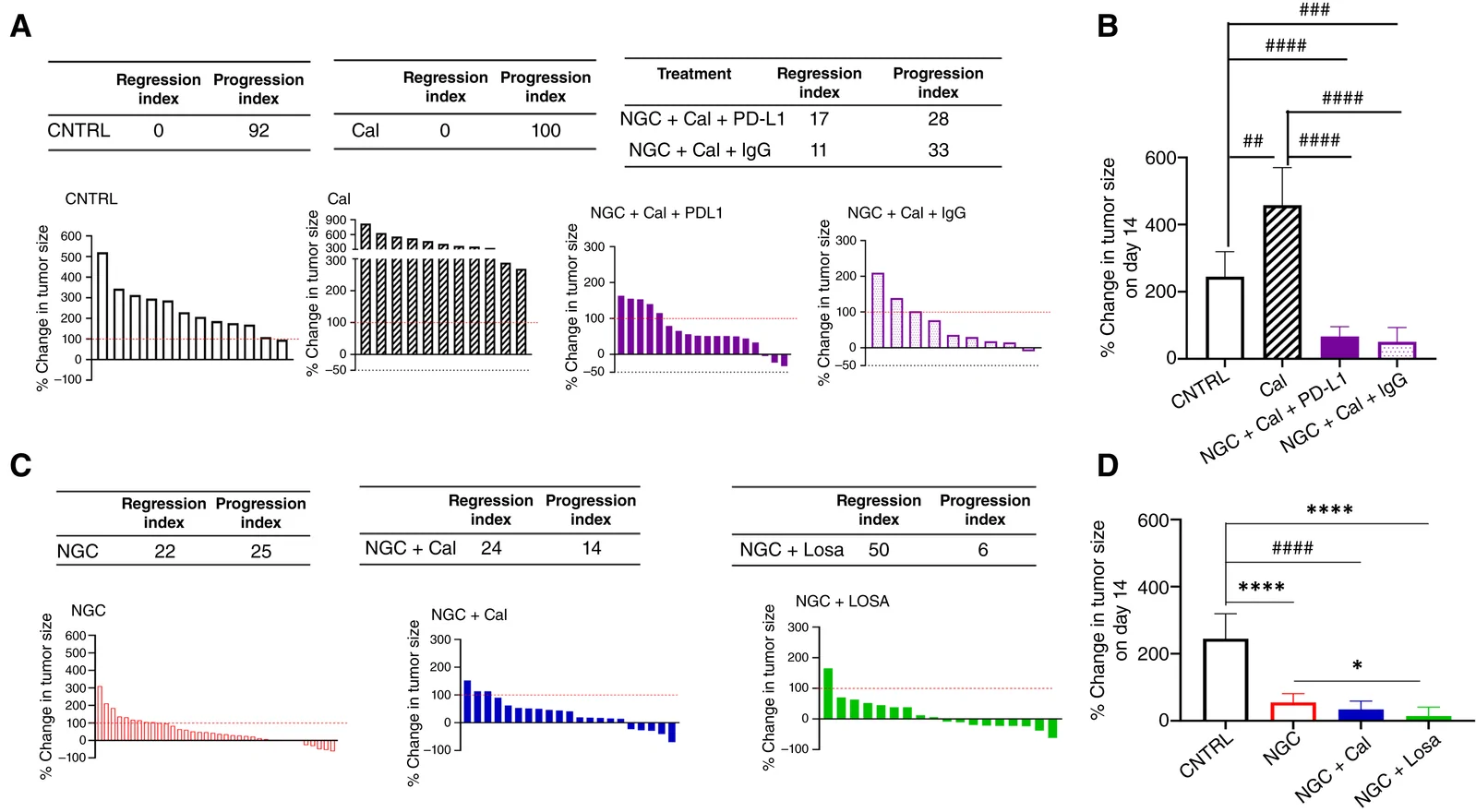
Tumor size change in preclinical trials. Waterfall plots of individual tumors and tumor size change on day 14 in the matched trial (**A** and **B**) and chemo–stromal trial (**C** and **D**). For each treatment group, a regression index is calculated as the percentage of tumors in which sizes were reduced compared with baseline (bars of negative values in waterfall plots), whereas a progression index is calculated as the percentage of tumors that doubled their volume (marked by the red dotted line). Sample size: control (CNTRL), *n* = 12; Cal, *n* = 11; NGC + Cal + PD-L1, *n* = 18; NGC + Cal + IgG, *n* = 9; NGC, *n* = 36; NGC + Cal, *n* = 21; NGC + LOSA, *n* = 18. *, *P* < 0.05; ****, *P* < 0.0001; Mann–Whitney test with two-tailed *P* value. ^##^, *P* < 0.01; ^###^, *P* < 0.001; ^####^, *P* < 0.0001; two-tailed *t* test (details in the “Statistical analysis” section).

KPC mouse studies included the “matched trial” and “chemo–stromal trial” (Fig. 1A and B). Tumor response was summarized using waterfall plots (Fig. 2A and C), from which we derived a “regression index,” defined as the percentage of tumors smaller on day 14 than at baseline, and a “progression index,” defined as the percentage of tumors that doubled in volume over the 14-day trial period. Based on these indices, in the matched trial, NGC + Cal + PD-L1 outperformed NGC + Cal + IgG, suggesting a moderate antitumor contribution from PD-L1 blockade (Fig. 2A). This effect may be underestimated because NGC + Cal showed higher regression and lower progression indices than NGC + Cal + IgG, noting that IgG was intended as an isotype control (Fig. 2B). Cal monotherapy increased tumor progression. However, NGC + Cal outperformed NGC, so did NGC + Losa, as shown in the chemo–stromal trial, confirming enhanced antitumor efficacy in combined treatments (Fig. 2C). Tumor volume increased significantly during the trial period in all groups except the NGC + Losa-treated group, in which greater inhibition of tumor growth was obtained compared with NGC alone (*P* < 0.05, Fig. 2D), leading to minimal growth and higher predicted odds of tumor regression (Supplementary Fig. S3A and S3B).

For ADC and cell density analysis in the clinical trial, the tumor in patient #7 (arm A) had a 17% decrease in ADC from baseline and exhibited the lowest cell density in the tumor bed compared with a 17% ADC increase and higher cell density in patient #3 (arm B; Supplementary Fig. S2B). In KPC tumors, the matched experimental regimen also reduced ADC by 16% from baseline (*P* < 0.05, Supplementary Fig. S4A); overall, changes in ADC and tumor cell density (Supplementary Fig. S4B) were modest across treatment groups, yielding an inverse correlation between ADC and cell density when all tumors were pooled (Supplementary Fig. S4C).

### Changes in microvasculature, matrix collagen, and FAP signature

For DCE-MRI in patients, eight recruited subjects underwent baseline imaging before subtype assignment; the mean tumor K^trans^ was 0.29 ± 0.12 min^−1^ (*n* = 7), excluding patient #4 because the tumor was indistinct from the pancreas. In evaluable patients, K^trans^ maps showed greater spatial heterogeneity at baseline but became more uniform after treatment with increased average K^trans^ values, corresponding to smaller tumor size after treatment; CD31 staining of resected specimens provided exploratory MVD data although staining was not successful for patient #7 (Supplementary Fig. S5A–S5C).

**Figure 3.**
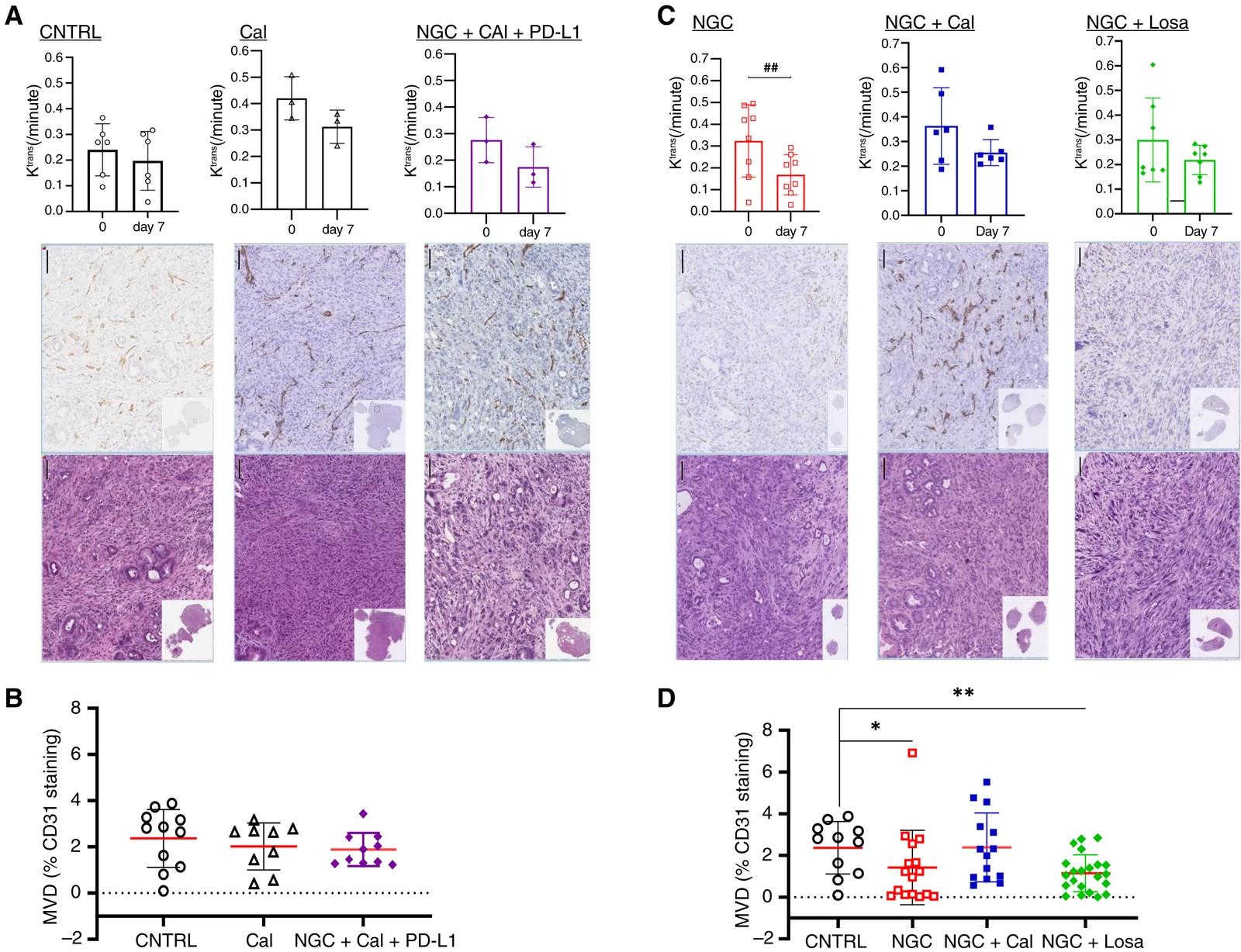
DCE metric over time and posttreatment MVD in KPC tumors. Plots of K^trans^ at baseline and day 7, CD31-stained micrographs, and MVD from treatment arms in the matched trial (**A** and **B**) and chemo–stromal trial (**C** and **D**). CD31-positive cells are stained brown. Sample size (MRI/IHC): CNTRL (control, *n* = 6/11), Cal (*n* = 3/9), NGC + Cal + PD-L1 (*n* = 3/10), NGC (*n* = 8/10), NGC + Cal (*n* = 6/8), and NGC + Losa (*n* = 7/22). Scale bars, 70 μm. ^##^, *P* < 0.01; two-tailed *t* test. *, *P* < 0.05; **, *P* < 0.01; Mann–Whitney test with two-tailed *P* value (details in “Statistical analysis”).

In KPC mice, the matched experimental regimen did not significantly change K^trans^ on day 7 or MVD on day 14 (Fig. 3A and B). In contrast, chemotherapy (NGC) reduced K^trans^ by 52% from baseline (*P* < 0.01) and decreased MVD relative to control (*P* < 0.05; Fig. 3C and D; Supplementary Fig. S5D). NGC + Cal and NGC + Losa partially restored K^trans^, but only NGC + Cal reversed the NGC-induced reduction in MVD. Thus, chemotherapy greatly affected microvascular function and density, whereas chemo–stromal combinations partially restored vascular function.

**Figure 4.**
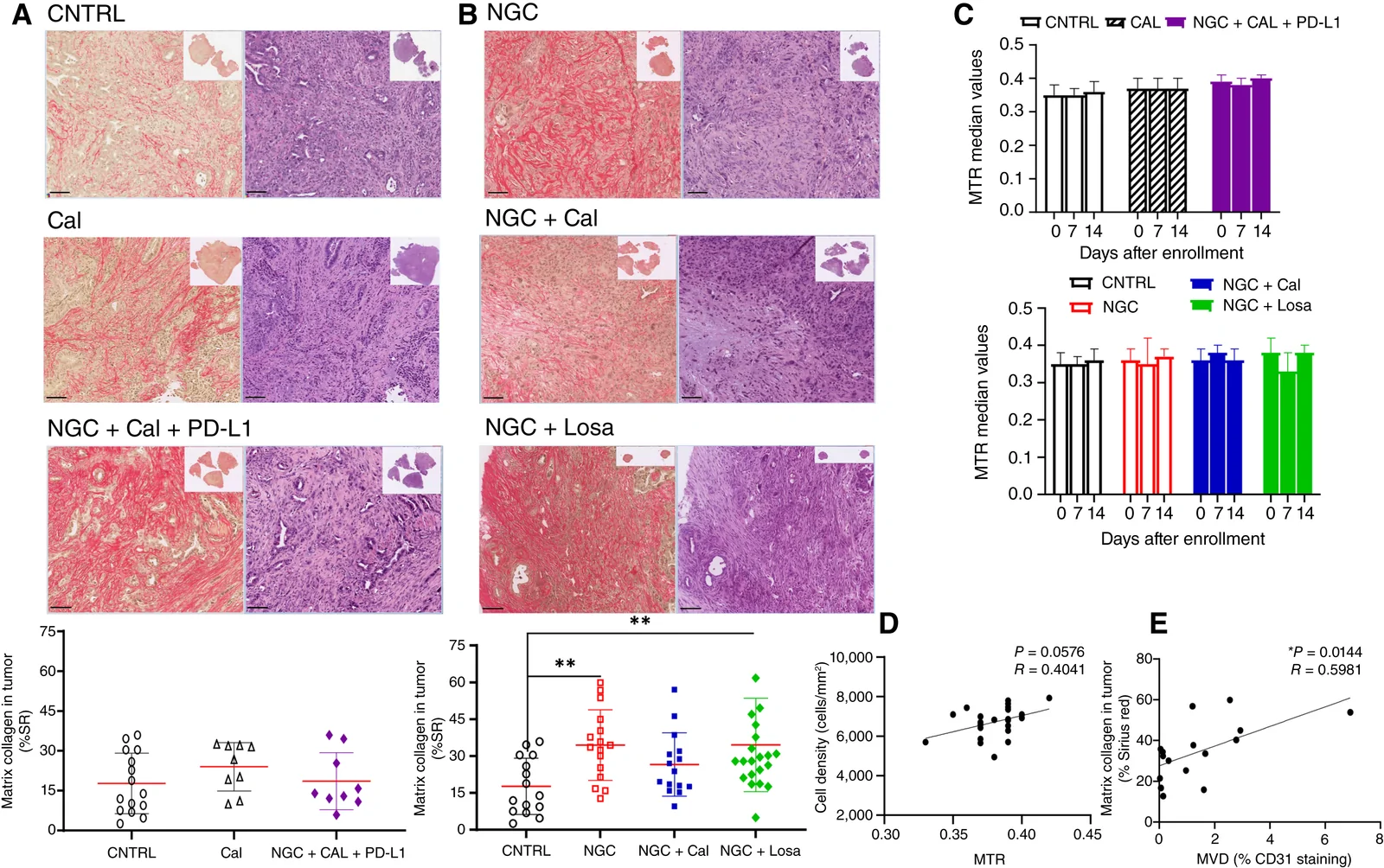
Matrix collagen level in KPC tumors. Micrographs of Sirius red (SR) staining of the tumor and posttreatment matrix collagen level estimated by %SR positive are presented for treatment arms in the matched trial (**A**) and the chemo–stromal trial (**B**). Time course of MTR in matched treatment arms (top, **C**) and the chemo–stromal trial (bottom, **C**). Correlation between MTR and cell density of the tumor (**D**). Correlation between matrix collagen and MVD (**E**). Scale bars, 70 μm. Sample size: control (CNTRL), *n* = 15; Cal, *n* = 9; NGC + Cal + PD-L1, *n* = 9; NGC, *n* = 16; NGC + Cal, *n* = 15; and NGC + Losa, *n* = 22. **, *P* < 0.01; Mann–Whitney test with two-tailed *P* value (details in the “Statistical analysis” section). The representative H&E image shown for the control group is from the same control tumor specimen shown in Fig. 3 but corresponds to a different tumor region, as indicated by the boxed area in the thumbnail image.

Sirius red staining of tumors from NCT03519308 showed relatively high but variable matrix collagen content (Supplementary Fig. S6). In KPC tumors, matrix collagen level did not change after the matched experimental regimen compared with control (16% vs. 14%, *P* = 0.856, Fig. 4A); similarly, Cal alone or Cal + NGC did not increase collagen above control (Fig. 4A and B). By contrast, NGC significantly increased collagen deposition between tumor nests (*P* < 0.01 vs. control), so did NGC + Losa (Fig. 4B). The MTR imaging marker did not change significantly during treatment, with a moderate correlation with cell density (*P* = 0.0576; Fig. 4C and D). Interestingly, a positive correlation between Sirius red and MVD was observed only in NGC-treated tumors (r = 0.5981, *P* = 0.0144; Fig. 4E), but not in other treatment groups or pooled data, suggesting that collagen may have context-dependent effects, including possible protection of neovasculature from chemotherapy-induced injury.

**Figure 5.**
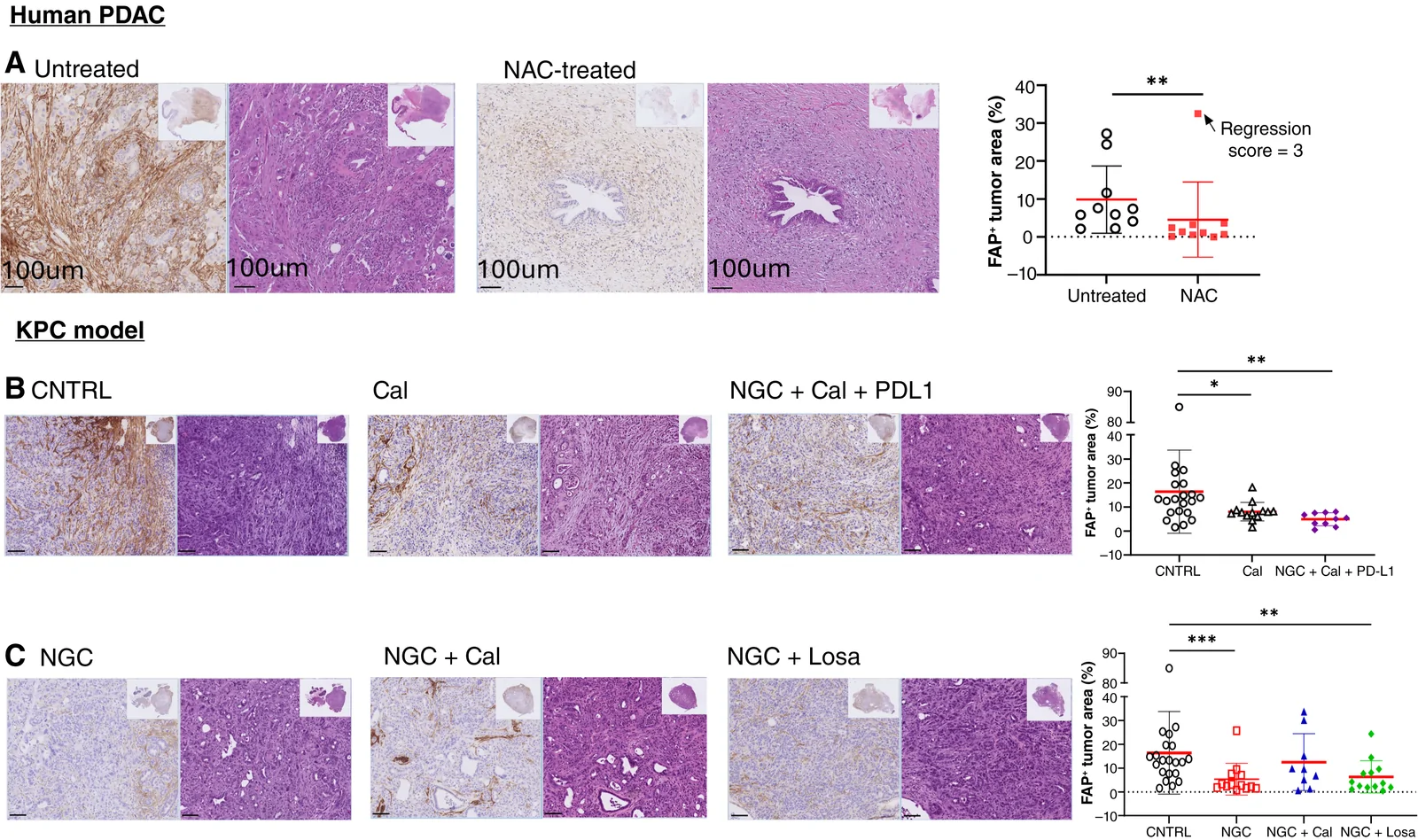
FAP in human PDAC surgical specimens and KPC tumors. FAP-stained micrographs and percentage of FAP staining in surgically resected PDAC tumors from patients who had NAC (*n* = 10) or were untreated (*n* = 10) before surgery (**A**). FAP-stained micrographs and quantifications from KPC tumors harvested after treatment from mice enrolled in the matched trial (**B**) and chemo–stromal trial (**C**). Sample size of KPC model: control (CNTRL), *n* = 21; Cal, *n* = 13; NGC + Cal + PD-L1, *n* = 10; NGC, *n* = 13; NGC + Cal, *n* = 9; and NGC + Losa, *n* = 13. Scale bars, 70 μm or as indicated. *, *P* < 0.05; **, *P* < 0.01; ***, *P* < 0.001. Mann–Whitney test with two-tailed *P* value (details in the “Statistical analysis” section).

Because FAP staining was not included in the NCT03519308 protocol, we analyzed PDAC surgical specimens from the biobank (IRB 853345). These specimens were collected from patients who underwent NAC (*n* = 10) or were untreated prior to surgery (*n* = 10). FAP staining was significantly lower after NAC (*P* < 0.01), whereas one high-FAP outlier had a regression score of 3, suggesting that this patient did not respond to NAC (Fig. 5A). In KPC tumors, the matched experimental regimen reduced FAP significantly (*P* < 0.01), so did Cal monotherapy (*P* < 0.05, Fig. 5B); NGC induced a remarkable reduction of FAP (*P* < 0.001, Fig. 5B and C), so did NGC + Losa (*P* < 0.01), but not after NGC + Cal, likely due to relatively larger variations and smaller sample size.

### Changes in KPC tumor transcriptome by single-cell RNA-seq analysis

**Figure 6.**
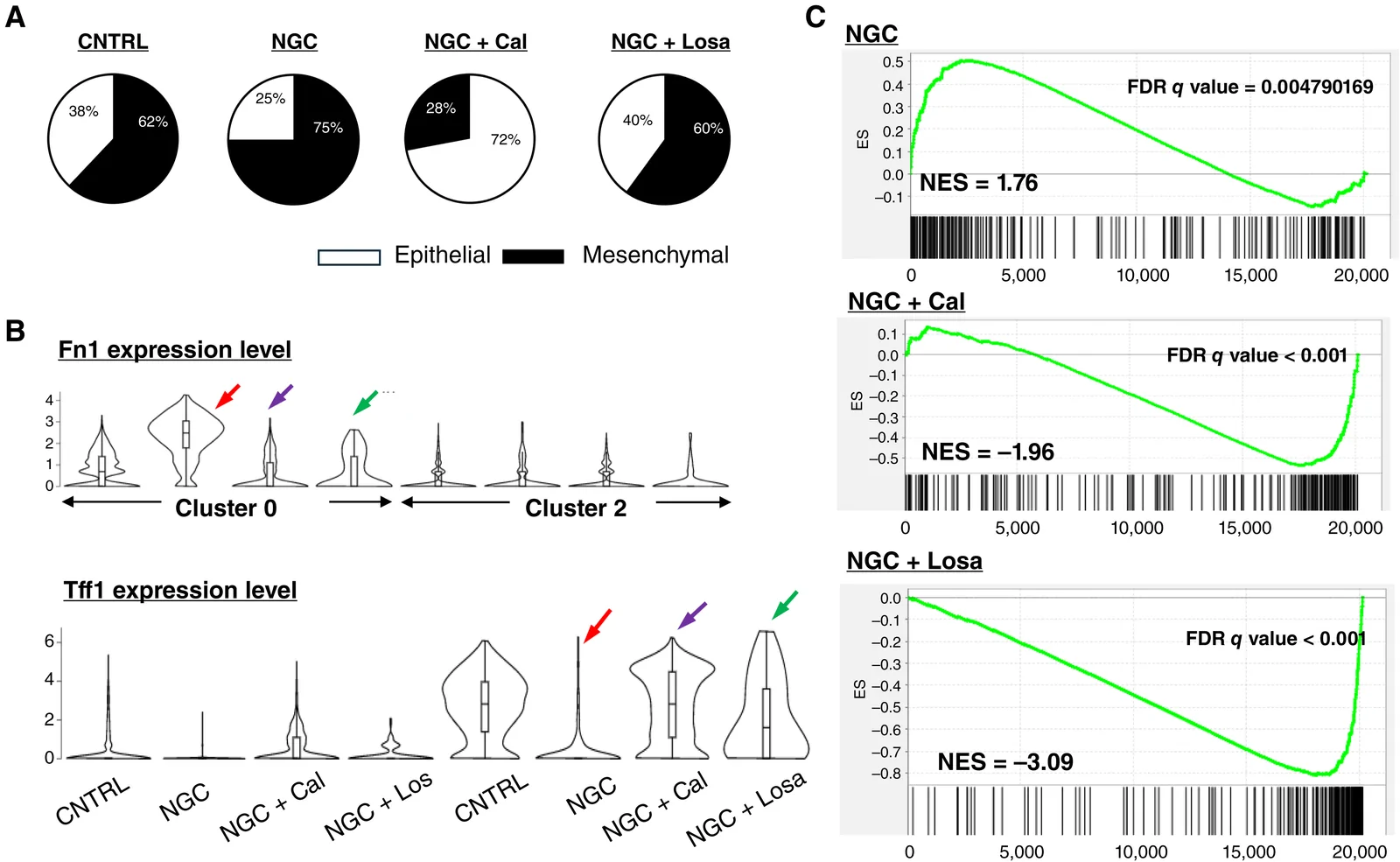
Single-cell RNA-seq analysis of KPC tumors. Fraction of epithelial versus basal/mesenchymal subtype cancer cells in tumors after specified treatment (**A**). Violin plots of the basal subtype signature gene Fn1 and the epithelial signature gene Tff1 expression of samples (**B**). Gene set enrichment analysis revealed EMT activation after NGC but not after NGC + Cal or NGC + Losa treatment (**C**). Sample size: Two tumors from each treatment group were combined for single-cell RNA-seq analysis. ES, enrichment score; NES, normalized enrichment score.

Exploratory single-cell RNA-seq was performed in KPC tumors. Pooled tumors from control, NGC, NGC + Cal, and NGC + Losa groups were analyzed. Uniform Manifold Approximation and Projection for Dimension Reduction identified cancer-cell, fibroblast, lymphocyte, and other stromal clusters (Supplementary Fig. S7A). Among cancer cells, NGC increased the mesenchymal-to-epithelial ratio (M/E = 75/25), whereas NGC + Cal reversed this pattern (M/E = 28/72; Fig. 6A), with similar results obtained using an independent analytic method (Supplementary Fig. S8). Consistently, NGC increased the expression of the mesenchymal marker Fn1 while decreasing the epithelial marker Tff1, whereas NGC + Cal and NGC + Losa attenuated these changes (Fig. 6B; Supplementary Fig. S9). Gene set enrichment analysis showed epithelial-to-mesenchymal transition (EMT) activation after NGC, but EMT was diminished after NGC + Cal or NGC + Losa (Fig. 6C).

Although all treatments reduced the cancer cell fraction by at least twofold versus control, NGC + Losa treatment almost completely depleted cancer cells and increased lymphocyte infiltration (Supplementary Fig. S7B and S7C). The improved tumor immune microenvironment can be exclusively associated with Losa because nab-paclitaxel, made of murine albumin (instead of human albumin), used in this study should not induce immune reaction, and NGC + Cal treatment did not increase lymphocyte infiltration relative to the control. Qualitatively, T-cell lineage and activation markers appeared stronger after NGC + Losa, and to a lesser extent after NGC + Cal, than after NGC or control treatment (Supplementary Fig. S7D). CAF transcriptome analysis revealed that NGC + Losa increased the expression of iCAF-associated genes while reducing myCAF-associated genes compared with controls (Supplementary Fig. S10).

## Discussion

This co-clinical study evaluated synthetic vitamin D combined with multiagent chemotherapy and an immune checkpoint inhibitor in a pilot perioperative trial and in the KPC mouse model. Although clinical accrual was limited, NCT03519308 confirmed the feasibility of repeated paricalcitol infusion with NGC plus immunotherapy. KPC model was then used to provide a statistically powered evaluation of the matched regimen and related chemo–stromal combinations. The matched KPC trial supported the antitumor activity of NGC + Cal + PD-L1 and suggested a moderate contribution from PD-L1 blockade. Cal monotherapy increased tumor progression, consistent with prior evidence that this agent can increase metastatic potential in basal PDAC models, underscoring the importance of subtype selection for VDR agonist-based strategies.

The 2-week preclinical treatment window captured significant stromal and vascular remodeling induced by chemotherapy and chemo–stromal combinations and demonstrated the value of DCE-MRI–derived markers in a co-clinical setting. A central finding is that NGC chemotherapy markedly remodels the PDAC stroma while also inducing features that may limit continued treatment response. NGC reduced microvascular function and density, increased matrix collagen deposition, suppressed FAP expression, and promoted EMT-associated transcriptional programs. The greater than 50% reduction in K^trans^ by day 7 is particularly important because K^trans^ reflects the rate at which contrast-agent molecules transfer from plasma to interstitial space; therefore, a reduction in K^trans^ serves as an imaging marker of impaired vascular delivery. In parallel, the enrichment of basal/mesenchymal cancer cells and EMT activation after NGC support a resistant phenotype and are consistent with prior observations that exposure to FOLFIRINOX promotes a basal subtype in human PDAC, whereas EMT activation and basal subtype enrichment contribute to chemoresistance (19). Together, these findings suggest that chemotherapy can be self-limiting by simultaneously promoting vascular and transcriptional adaptations that reduce chemosensitivity.

Cal and Losa both enhanced the antitumor effect of NGC but seemed to do so through distinct stromal mechanisms. NGC + Cal partially restored K^trans^, preserved MVD, prevented excess collagen accumulation, and attenuated NGC-induced EMT enrichment. These findings support the concept that rather than depleting stroma, VDR agonism promotes stromal normalization by shifting an inflammatory phenotype toward a more quiescent one, improving vascular function, and thereby maintaining chemosensitivity. Increased tumor vascularity and reduced inflammation were reported in PDAC biopsies from patients receiving paricalcitol treatment in combination with chemotherapy (28). Losa has been investigated as a stromal modulator in PDAC, with prior clinical studies showing feasibility and encouraging outcomes but no clear incremental benefit in a randomized phase II study (29, 30). In the KPC model, NGC + Losa produced the strongest tumor growth inhibition and increased lymphocyte infiltration while maintaining high matrix collagen levels. This pattern is consistent with Losa-mediated improvement in vascular patency through angiotensin receptor blockade and potential modulation of TGFβ-associated profibrotic and immunosuppressive pathways (31), thereby enhancing the tumor immune microenvironment, similar to what was observed in human PDAC studies (32).

FAP findings were consistent across human and mouse specimens. NAC markedly reduced FAP staining in human PDAC surgical specimens, and similar suppression was observed in KPC tumors after NGC-containing regimens. Because high FAP expression is associated with shortened overall survival, desmoplasia, immune exclusion, and recruitment of suppressive myeloid cells in human PDAC (33, 34), treatment-induced FAP reduction may reflect favorable stromal remodeling. The high-FAP outlier with poor regression after NAC raises the possibility that persistent FAP expression could identify treatment resistance, but this requires confirmation in larger clinical cohorts.

The imaging results highlight DCE-MRI as the most informative MRI marker in this study. Clinical and preclinical DCE-MRI protocols both used radial acquisition and reconstruction strategies, and baseline K^trans^ values were broadly concordant between patients and KPC tumors. DCE-MRI detected early chemotherapy-induced vascular deficiency and partial restoration by stromal-directed combinations, changes that would be difficult to assess serially by tissue-based MVD analysis. In contrast, ADC showed only modest treatment-related changes, likely because reduced cellularity, collagen deposition, inflammation, and compensatory proliferation can influence water mobility in opposing directions. MTR did not track collagen content in KPC tumors and seemed more sensitive to cellular macromolecular content (cell density) than to ECM collagen under the imaging protocol used here.

In the past 5 years, several clinical trials have investigated paricalcitol for patients with locally advanced or metastatic PDAC and confirmed its safety (28, 35–39) while also demonstrating the benefit of oral administration and a pharmacodynamic effect of paricalcitol (40). Importantly, other PDAC trials have suggested the efficacy of paricalcitol in combination with NGC, with or without nivolumab (38, 39). Together, findings from these trials and the present co-clinical study support continued evaluation of this stromal-directed strategy and should inform the design of larger efficacy trials.

## Supplementary Material

Supplementary Figure S1 Figure S1. Design of clinical trial (NCT03519308).

Supplementary Figure S2 Figure S2. Exploratory results from NCT03519308: Full-mount histology of surgically resected tumors, tumor ADC values and cellularity.

Supplementary Figure S3 Figure S3. Time course of tumor volume in KPC mice enrolled in preclinical trials and odds ratio analysis.

Supplementary Figure S4 Figure S4. Tumor ADC time course, cell density and their correlation in KPC mice.

Supplementary Figure S5 Figure S5. Exploratory results from NCT03519308: Ktrans maps of patients’ tumors and capillary density in surgically resected tumors in comparison with Ktrans maps from KPC mice.

Supplementary Figure S6 Figure S6. Exploratory results from NCT03519308: Matrix collagen content in surgically resected tumors.

Supplementary Figure S7 Figure S7. Uniform Manifold Approximation and Projection (UMAP), cell type distribution and T cell markers of KPC tumors.

Supplementary Figure S8 Figure S8. Using a different approach to confirm Figure 6A results.

Supplementary Figure S9 Figure S9. Transcriptome changes of cancer cells (cluster 0 and 2) in epithelial and basal panel genes in CNTRL, NGC, NGC combined with Cal or Losa, respectively.

Supplementary Figure S10 Figure S10. Transcriptome changes in cancer associated fibroblasts, CAFs after chemotherapy and its combination with calcipotriol or losartan.

Supplementary Table S1 Table S1. Meta data of recruited patients in NCT 03519308. Enrolled ones are bolded.

Supplementary Table S2 Table S2. Patient representativeness of the clinical trial

Supplementary Methods Supplementary Methods

## Data Availability

Deidentified clinical MRI and histopathology data from participants enrolled in NCT03519308 and representative preclinical MRI data are publicly available via the NCI-funded Co-Clinical Imaging Resource Program at the following link: https://pennpancreaticcancerimagingresource.github.io/imagedata.html. Remaining preclinical MRI and preclinical histopathology data are available from the corresponding authors upon reasonable request. Single-cell RNA-seq data are deposited in an International Nucleotide Sequence Database Collaboration repository and can be accessed with BioProject accession code PRJNA1509429 and Sequence Read Archive accessions (SRR40066542, SRR40066543, SRR40066544 and SRR40066545).
