# Supplementary material for: Co-Clinical Evaluation of Synthetic Vitamin D and Chemotherapy in Pancreatic Cancer by Integrated Imaging and Molecular Analyses
Source: Cancer Res Commun. 2026 Aug 25;6(8):1983–92. doi: 10.1158/2767-9764.CRC-26-0443 (PMC13505481; doi:10.1158/2767-9764.CRC-26-0443)
Supplement: Supplementary file 1 [file crc-26-0443_supplementary_methods.pdf]

Supplementary Methods

Clinical studies

Clinical trial design

A Randomized Pilot Study of Perioperative Nivolumab and Paricalcitol to Target the Microenvironment in Resectable Pancreatic Cancer (NCT03519308) was approved by the University of Pennsylvania Institutional Review Board. The trial enrolled patients with resectable PDAC after endoscopic core biopsy and molecular subtyping. Only patients with epithelial-subtype PDAC were eligible for enrollment; while subtype results were pending, patients received one cycle of chemotherapy. The chemotherapy backbone consisted of nab-paclitaxel, gemcitabine, and cisplatin (NGC). Patients were assigned to NGC plus nivolumab with weekly paricalcitol or NGC plus nivolumab without paricalcitol. Research MRI was performed at baseline before neoadjuvant therapy and after completion of therapy but before surgical resection. NGC was selected as the chemotherapy backbone because, at the time of study design, adding cisplatin to nab-paclitaxel and gemcitabine had shown high response rates in advanced PDAC (1); the similar effectiveness of nab-paclitaxel/gemcitabine and FOLFIRINOX in the perioperative setting was reported later in SWOG S1505 (2).

Clinical MRI acquisition and analysis

Patients recruited to NCT03519308 for evaluation consented separately for the MRI. Clinical MRI was performed on a Siemens Avanto 1.5T system using torso and spine RF coil arrays. After localizers and T2-weighted imaging, diffusion-weighted imaging was acquired using a single-shot EPI sequence with 90 degree flip angle, TE/TR = 79/2400 ms, echo spacing = 0.69 ms, field of view = 380 x 380 mm2, matrix size = 192 x 192, slice thickness = 9 mm, 1 mm interslice gap, 16 slices, receiver bandwidth = 1735 Hz/pixel, and b-values of 0, 50, 100, 200, 500, 800, 1200, and 2000 s/mm2. Dynamic contrast-enhanced MRI was acquired using a three-dimensional stack-of-stars spoiled radial sequence with in-plane golden-angle view increment and phase encoding along the slice direction (3). DCE-MRI parameters were 25 degree flip angle, TE/TR = 1.74/3.66 ms, field of view = 380 x 380 mm2, matrix size = 256 x 256, slice thickness = 5 mm, 32 slices, receiver bandwidth = 100 Hz/pixel, and 3520 view angles. Dotarem was injected at 0.2 mL/kg beginning 1.5 minutes after dynamic acquisition. Before DCE-MRI, intrinsic tumor T1 was estimated using the same radial sequence with flip angles of 4, 10, 15, and 25 degrees. K-space weighted image contrast reconstruction was used for DCE-MRI, yielding an effective temporal resolution of 5.234 s and 64 temporal frames (4).

Tumor size was measured from long-axis and short-axis dimensions on T2-weighted MRI according to RECIST-based criteria. Percent change in long-axis dimension from baseline to post-treatment was reported for each patient. Tumor ADC was calculated by fitting diffusion-weighted signal intensities to a single-exponential decay model. DCE-MRI was analyzed using standard Tofts and shutter-speed models implemented in Interactive Data Language. Tumor T1 was calculated by fitting multiple flip-angle radial acquisitions to the steady-state gradient-echo equation. Image intensity was converted to gadolinium concentration assuming intrinsic blood T1 = 1200 ms and Dotarem relaxivity = 3.6 mL/mM/ms. Dynamic fitting used the IDL "amoeba" downhill simplex function to generate parametric maps, including K^{trans} and V_e (5) (6).

Clinical immunohistochemistry

Surgically resected tumors from patients enrolled in NCT03519308 were processed by the Clinical Pathology Laboratory, Department of Pathology, Abramson Cancer Center, University of Pennsylvania. Adjacent formalin-fixed paraffin-embedded sections were stained for H&E, Sirius Red, and CD31.

Additional PDAC surgical specimens for FAP staining were obtained from the University of Pennsylvania biobank (IRB Protocol #853345), including tumors from patients who received neoadjuvant chemotherapy before surgery (NAC, n=10) and from untreated patients who underwent upfront surgery (n=10).

Adjacent sections from these specimens were stained for H&E and fibroblast activation protein (FAP). Stained sections were scanned at 40x magnification using an Aperio ScanScope CS2 and analyzed by a gastrointestinal pathologist (EEF) blinded to treatment information.

Preclinical studies

Materials

Gemcitabine, cisplatin, losartan and ProHance®, a Gd-based contrast agent used in the clinic were all purchased from the Pharmacy of the Hospital of University of Pennsylvania. A synthetic derivative of calcitriol (1,25 OH-cholecalciferol), Calcipotriol, is a biologically active form of vitamin D and was used for administration in mice (7). Pharmaceutical grade of calcipotriol (catalog 270010) and murine serum albumin (catalog 502036442) were purchased from Fisher Scientific (Waltham, MA, USA), paclitaxel (Catalog P-9600) from LC Laboratories (Woburn, MA, USA), anti-PDL1 mAb (clone 10F.9G2, catalog BP0101) and the control immunoglobulin G (IgG, clone LTF-2, catalog BE0090) were purchased from Bio-X Cell (Lebanon, NH, USA).

Manufacturing of murine nab-paclitaxel

To avoid potential immune effects from human albumin in clinical nab-paclitaxel, a murine albumin formulation of nab-paclitaxel was prepared using a published protocol (8). Paclitaxel (30 mg) was dissolved in 0.55 mL chloroform and 0.05 mL ethanol, then added to 30 mL PBS containing 1% w/v murine albumin pre-saturated with 1% chloroform. The mixture was emulsified using a high-pressure homogenizer at approximately 15,000 psi for at least six cycles. Chloroform was removed by rotary evaporation, and the solution was lyophilized. The resulting powder was stored at -20°C, reconstituted in PBS to 5 mg paclitaxel/mL, and injected shortly after preparation.

In vivo studies and preclinical trial design

All animal procedures were approved by the University of Pennsylvania Institutional Animal Care and Use Committee. A genetically engineered KPC mice (KrasG12D:Trp53R172H:Pdx1-Cre) were bred at the Mouse Hospital of the Pancreatic Cancer Research Center. Tumor screening began at 11 weeks of age by weekly abdominal palpation followed by ultrasound examination using a Vevo 2100 system. Once transferred to the study, mice underwent MRI to confirm tumor size before enrollment.

Mice with MRI-estimated tumor volume of 104 ± 40 mm3 were enrolled. In the first preclinical trial, the regimen matched to the experimental clinical arm, NGC plus calcipotriol and anti-PDL1 antibody, was tested; calcipotriol monotherapy was also evaluated. In the second trial, NGC alone was compared with NGC plus calcipotriol or NGC plus losartan. Both male and female mice were used and randomized to treatment groups. All treatments were administered intraperitoneally according to the schedule in **Fig. 1**. NGC was administered on Days 0 and 7. Anti-PDL1 antibody, IgG control antibody, and stromal-directed agents were administered more frequently during the two-week trial. Control mice were untreated. MRI was performed on Days 0, 7, and 14 for T2-weighted imaging, diffusion-weighted MRI, and MTR imaging; DCE-MRI was performed on Days 0 and 7. When MRI and treatment occurred on the same day, MRI was performed before treatment.

In vivo MRI

General anesthesia was induced and maintained with 1-3% isoflurane in air delivered through a nose cone. For DCE-MRI, a tail-vein catheter with extension tubing preloaded with ProHance diluted 50-fold in PBS to 10 mM gadolinium was placed before imaging. Mice were positioned in a 35 x 40 mm quadrature birdcage RF coil. Respiration and temperature were monitored using a pneumatic pillow and rectal temperature probe, and core temperature was maintained at 37 ± 0.2°C with warm air.

MRI was performed on a 9.4T Bruker Avance III system equipped with 12 cm inner-diameter gradients capable of 40 G/cm. T2-weighted images spanning the tumor were acquired using a TurboRARE protocol with effective TE = 30 ms, TR = 1.8 s, echo train length = 8, matrix = 64 x 64, 16 slices, field of view = 32 x 32 mm, slice thickness = 0.5 mm, four averages, and approximately 2 minutes acquisition time. The T2-weighted image geometry was passed to DW-MRI, DCE-MRI, and MTR acquisitions.

DW-MRI used a radial k-space sampling scheme to reduce respiratory motion artifacts during free breathing (9,10). Acquisition parameters were diffusion time (Δ) = 14 ms, diffusion gradient duration (δ) = 9 ms, b-values of 10, 535, 1070, 1479, and 2141 s/mm2, TE/TR = 28.6/750 ms, matrix = 64 x 403, one average, 50 kHz bandwidth, fat saturation, and approximately 25 minutes total acquisition time. The detailed DWI protocol is available at protocols.io (dx.doi.org/10.17504/protocols.io.kxygxp6jol8j/v1).

For DCE-MRI, a three-dimensional stack-of-stars sequence was used for B1 mapping by actual flip angle imaging, T1 mapping by variable flip angle imaging, and golden-angle dynamic contrast acquisition (3). Two minutes after starting the DCE series, 0.2 mL diluted ProHance was injected manually over 10 s. All DCE components used acquisition matrix = 64 x 201 x 16 and field of view = 32 x 32 x 8 mm3. The detailed DCE protocol is available at protocols.io (dx.doi.org/10.17504/protocols.io.81wgb6pnolpk/v1).

For MTR imaging, a magnetization-prepared three-dimensional gradient-echo sequence was acquired with field of view = 32 x 32 x 8 mm, matrix = 64 x 64 x 16, TE/TR = 2.8/5.7 ms, flip angle = 5 degrees, four averages, and an 18 s 2.5 microtesla saturation pulse applied at 4 kHz for the MTon image and 250 kHz off-resonance for the reference image. Each saturation pulse was followed by centric phase-encoded full-plane k-space acquisition.

MRI Data Analytics

The tumor volume was determined by manually tracing the tumor boundary on T2W images to generate tumor Region Of Interest (ROI), summing the areas from all ROIs and multiplying the result by the slice thickness. DWI, DCE and MTR metrics were estimated over the same ROIs. To produce waterfall plots, the percentage (%) change in tumor volume between Day0 (baseline) and Day14 was plotted for each mouse.

DW-MRI metrics: Radial k-space data were reconstructed to images using custom python codes (https://github.com/PennPancreaticCancerImagingResource/DWIProcessing). The reconstructed images were processed with pixel-wise least squares fit to Equation [1] using all five b-values as described previously (10), where S_0, *ADC*, and *KI* (kurtosis index) are the fitting parameters: S_0 is the signal intensity in the absence of diffusion weighting and *KI* is a constant that accounts for contributions from water molecules undergoing slow hence non-gaussian diffusion to the observed signal *S(b).*

$$S(b) = S_0 \exp\{-b \times ADC\} + KI \quad [1]$$

DCE-MRI metrics: Reconstruction of radial k-space data using k-space weighted image contrast (KWIC) method and analysis of the DCE series to derive the DCE metrics were described in our prior studies (3): K^{trans} (rate constant of transferring unit volume of contrast agent from capillaries to interstitial space per minute), V_e (extracellular and extravascular volume fraction). Pixel-wise K^{trans} and V_e were generated for all pixels in the tumor ROIs.

MTR metrics: MTR value was calculated pixel-wise by equation [2] to generate MTR maps from tumor ROIs:

$$MTR = (Reference - MT_{on})/Reference \quad [2],$$

where the MT_{on} and *Reference* image were acquired with and without saturation of motion-restricted water, respectively.

For MRI metrics (ADC, KI, K^{trans}, V_e, MTR), the median value is estimated from pixels in regions of interest (ROIs) of each tumor, while the mean +/- standard deviation and median +/- 95% confidence interval (CI) were calculated for each treatment group.

Ex vivo studies

Immunohistochemistry and analysis

Formalin-fixed paraffin-embedded KPC tumor sections were stained with H&E, CD31, Sirius Red, or FAP, with one section per stain per tumor, by the pathology cores at the Children's Hospital of Philadelphia or the University of Pennsylvania (10). Stained sections were scanned at 40x magnification using an Aperio ScanScope CS2. A gastrointestinal pathologist (EEF) blinded to treatment information reviewed the sections. To avoid field-selection bias, whole-section analysis was performed in QuPath using pixel-classifier methods (10). The tumor bed was segmented, and non-tumor regions, including lymph nodes, pancreas, and chronic pancreatitis, were excluded.

Single-cell RNA sequencing and analysis

Single-cell RNA sequencing analysis was applied to tumors harvested from KPC mice in control, NGC, NGC plus calcipotriol, and NGC plus losartan groups. It was considered exploratory as two tumors from each treatment group were pooled. Viable cell collection included tumor dissociation, red blood cell lysis, debris clearance, and dead-cell removal. Cells were suspended in 0.04% PBS at more than 200,000 cells per 100 microliters and submitted to the Center for Applied Genomics at the Children's Hospital of Philadelphia. Libraries were prepared using the 10x Genomics Chromium Single Cell 3' Reagent Kit v3 according to the manufacturer's instructions, indexed using the Chromium dual index kit, pooled, and sequenced on an Illumina NovaSeq 6000 in paired-end dual-index mode. Sequencing targeted 20,000 reads per cell.

Data were processed using Cell Ranger v6.1.2 for demultiplexing, alignment to the mm10 transcriptome, and feature-barcode matrix generation. Filtered matrices were integrated in Seurat v4, followed by SCTransform v2 normalization and unsupervised clustering. Automated cell-type assignments were performed in Bioconductor (RRID:SCR_006442) version3.16 using SingleR with celldex databases. Cirrocumulus was used for interactive data exploration and visualization. Heatmaps and violin plots were generated in Cirrocumulus. Epithelial, basal/mesenchymal, and CAF-related gene sets were selected from published studies (11-14).

Statistical analysis

Data are presented as mean ± standard deviation unless otherwise specified. Statistical analyses were performed using GraphPad Prism version 10.1.2 or R version 4.3.1. Statistical significance was defined as alpha = 0.05. Shapiro-Wilk testing was used to assess normality for MRI metrics, including tumor size, ADC, KI, K^{trans}, V_e, and MTR. Normally distributed MRI variables were analyzed using two-tailed t tests, and non-normally distributed variables were analyzed using Mann-Whitney tests. Immunohistochemistry metrics were analyzed using Mann-Whitney tests. Logistic regression was used to evaluate the likelihood of tumor regression, defined as reduction in tumor size from baseline to Day 14, comparing NGC with NGC plus calcipotriol and NGC plus losartan.

References

1. Jameson GS, Borazanci E, Babiker HM, Poplin E, Niewiarowska AA, Gordon MS*, et al.* Response Rate Following Albumin-Bound Paclitaxel Plus Gemcitabine Plus Cisplatin Treatment Among Patients With Advanced Pancreatic Cancer: A Phase 1b/2 Pilot Clinical Trial. JAMA oncology **2019**;6:125-32
2. Ahmad SA, Duong M, Sohal DPS, Gandhi NS, Beg MS, Wang-Gillam A*, et al.* Surgical Outcome Results From SWOG S1505: A Randomized Clinical Trial of mFOLFIRINOX Versus Gemcitabine/Nab-paclitaxel for Perioperative Treatment of Resectable Pancreatic Ductal Adenocarcinoma. Ann Surg **2020**;272:481-6
3. Pickup S, Romanello M, Gupta M, Song HK, Zhou R. Dynamic Contrast-Enhanced MRI in the Abdomen of Mice with High Temporal and Spatial Resolution Using Stack-of-Stars Sampling and KWIC Reconstruction. Tomography : a journal for imaging research **2022**;8:2113-28
4. Song HK, Dougherty L. k-space weighted image contrast (KWIC) for contrast manipulation in projection reconstruction MRI. Magn Reson Med **2000**;44:825-32
5. Cao J, Pickup S, Rosen M, Zhou R. Impact of Arterial Input Function and Pharmacokinetic Models on DCE-MRI Biomarkers for Detection of Vascular Effect Induced by Stroma-Directed Drug in an Orthotopic Mouse Model of Pancreatic Cancer. Mol Imaging Biol **2023**;25:638-47
6. Tofts PS. Modeling tracer kinetics in dynamic Gd-DTPA MR imaging. J Magn Reson Imaging **1997**;7
7. Sherman Mara H, Yu Ruth T, Engle Dannielle D, Ding N, Atkins Annette R, Tiriac H*, et al.* Vitamin D Receptor-Mediated Stromal Reprogramming Suppresses Pancreatitis and Enhances Pancreatic Cancer Therapy. Cell **2014**;159:80-93
8. Desai NP, Chunlin Tao C, Yang A, Louie L, Zheng T, Yao Z*, et al.* Protein stabilized pharmacologically active agents, methods for the preparation thereof and methods for the use thereof. US Patent 5,916,596A **1999**
9. Cao J, Song HK, Yang H, Castillo V, Chen J, Clendenin C*, et al.* Respiratory Motion Mitigation and Repeatability of Two Diffusion-Weighted MRI Methods Applied to a Murine Model of Spontaneous Pancreatic Cancer. Tomography : a journal for imaging research **2021**;7:66-79
10. Romanello Joaquim M, Furth EE, Fan Y, Song HK, Pickup S, Cao J*, et al.* DWI Metrics Differentiating Benign Intraductal Papillary Mucinous Neoplasms from Invasive Pancreatic Cancer: A Study in GEM Models. Cancers **2022**;14:4017
11. Hosein AN, Huang H, Wang Z, Parmar K, Du W, Huang J*, et al.* Cellular heterogeneity during mouse pancreatic ductal adenocarcinoma progression at single-cell resolution. JCI Insight **2019**;4:e129212
12. Porter RL, Magnus NKC, Thapar V, Morris R, Szabolcs A, Neyaz A*, et al.* Epithelial to mesenchymal plasticity and differential response to therapies in pancreatic ductal adenocarcinoma. Proceedings of the National Academy of Sciences **2019**;116:26835-45
13. Chen Y, Kim J, Yang S, Wang H, Wu CJ, Sugimoto H*, et al.* Type I collagen deletion in αSMA(+) myofibroblasts augments immune suppression and accelerates progression of pancreatic cancer. Cancer Cell **2021**;39:548-65.e6
14. Elyada E, Bolisetty M, Laise P, Flynn WF, Courtois ET, Burkhart RA*, et al.* Cross-Species Single-Cell Analysis of Pancreatic Ductal Adenocarcinoma Reveals Antigen-Presenting Cancer-Associated Fibroblasts. Cancer Discov **2019**;9:1102-23
