# Supplementary material for: Co-Clinical Evaluation of Synthetic Vitamin D and Chemotherapy in Pancreatic Cancer by Integrated Imaging and Molecular Analyses
Source: Cancer Res Commun. 2026 Aug 25;6(8):1983–92. doi: 10.1158/2767-9764.CRC-26-0443 (PMC13505481; doi:10.1158/2767-9764.CRC-26-0443)
Supplement: Supplementary file 2 [file crc-26-0443_supplementary_table_s1_suppst1.pdf]

## SI Table-1

Demographic data of recruited patients and treatment outcome (**bolded** are enrolled to NCT 03519308 )

| Patient # | Age | Gender | Race | Ethnicity | PDA subtype | Enrolled or excluded | MRI[b] | Surgery |
|---|---|---|---|---|---|---|---|---|
| 1 | 57 | male | Caucasian | Non-Hispanic | Quasimesenchymal | excluded | PRE-Tx only | NA |
| 2 | 54 | female | African American | Non-Hispanic | Insufficient tissue | excluded | PRE-Tx only | NA |
| **3** | **55** | **male** | **African American** | **Non-Hispanic** | **Epithelial** | **Arm-B** | **2 visits** | **Laparoscopic distal pancreatectomy** |
| **4** | **60** | **male** | **Caucasian** | **Hispanic** | **Epithelial** | **Arm-B** | **2 visits** | **Whipple** |
| 5 | 76 | female | Caucasian | Non-Hispanic | Insufficient tissue | excluded | PRE-Tx only | NA |
| 6 | 71 | female | Caucasian | Non-Hispanic | Insufficient tissue | excluded | PRE-Tx only | NA |
| **7** | **56** | **female** | **Caucasian** | **Non-Hispanic** | **Epithelial** | **Arm-A** | **2 visits** | **Laparoscopic distal pancreatectomy and splenectomy with G tube and J tube placement.** |
| 8 | 48 | female | Caucasian | Non-Hispanic | Insufficient tissue | excluded | None | NA |
| 9 | 68 | female | Caucasian | Non-Hispanic | Quasimesenchymal | excluded | PRE-Tx only | NA |

**Supplementary Table S1** **Meta data of recruited patients in NCT 03519308**. Enrolled ones are **bolded**.
