# Supplementary material for: Co-Clinical Evaluation of Synthetic Vitamin D and Chemotherapy in Pancreatic Cancer by Integrated Imaging and Molecular Analyses
Source: Cancer Res Commun. 2026 Aug 25;6(8):1983–92. doi: 10.1158/2767-9764.CRC-26-0443 (PMC13505481; doi:10.1158/2767-9764.CRC-26-0443)
Supplement: Supplementary file 3 [file crc-26-0443_supplementary_table_s2_suppst2.pdf]

Supplementary Table 2. Representativeness of Study Participants	
**Cancer type(s)/subtype(s)/stage(s)/ condition**	Resectable pancreatic ductal adenocarcinoma (PDAC) with epithelial molecular subtype. Subtype assignment was performed through analysis of pretreatment biopsy tissue; patients with quasimesenchymal subtype or insufficient tissue for subtype determination were not enrolled.
**Considerations related to:**	
**Sex**	PDAC affects both men and women and is slightly more common in men. In the United States, 2026 pancreatic cancer estimates include 35,190 cases in men and 32,340 cases in women. In this study, 9 patients were recruited/screened (3 male, 6 female), and 3 epithelial-subtype patients were enrolled (2 male, 1 female). The enrolled sex distribution should be interpreted cautiously because of the very small sample size.
**Age**	PDAC is predominantly diagnosed in older adults; SEER reports a median age at diagnosis of 71 years, with the largest proportion of new cases diagnosed at 65-74 years. The recruited/screened cohort had a median age of 57 years (range, 48-76), and the enrolled cohort had a median age of 56 years (range, 55-60). Thus, the enrolled population was younger than the broader PDAC population, likely reflecting the requirement for resectable disease, pretreatment biopsy-based subtype assignment, serial MRI, protocol therapy, and surgery.
**Race/ethnicity**	SEER data show racial and ethnic differences in pancreatic cancer incidence, with the highest age-adjusted incidence among non-Hispanic Black individuals. In this study, the recruited/screened cohort included 7 Caucasian and 2 African American patients; 8 were non-Hispanic and 1 was Hispanic. The enrolled cohort included 2 Caucasian patients and 1 African American patient; 2 were non-Hispanic and 1 was Hispanic. Because only 3 patients were enrolled, the study cannot evaluate racial or ethnic differences in feasibility, efficacy, toxicity, imaging findings, or outcome.
**Geography**	In 2026, an estimated 67,530 new pancreatic cancer cases and 52,740 deaths are expected in the United States. This was a single-institution, academic-center study, and the enrolled cohort represents patients who were referred, biopsied, molecularly classified, treated, imaged, and surgically managed within that setting. Therefore, the cohort may not represent patients treated primarily in community settings or those facing referral, travel, insurance, socioeconomic, or access barriers.
**Other considerations**	This trial was intentionally restricted to resectable epithelial-subtype PDAC. Among 9 recruited/screened patients, 3 were enrolled; 6 were excluded because the biopsy showed quasimesenchymal subtype (n=2) or had insufficient tissue for subtype assignment (n=4). This design enriched the study for a biologically and clinically selected subgroup and excludes those with locally advanced or metastatic disease, non-epithelial subtype, inadequate biopsy tissue, poor performance status, or medical conditions precluding protocol therapy, or surgery.
**Overall representativeness of this study**	The study should be interpreted as a highly selected, hypothesis-generating cohort of biopsy-classified, epithelial-subtype, resectable PDAC. The enrolled patients (n=3) were ages 55, 56, and 60; 2 were male and 1 was female; 2 were Caucasian and 1 was African American; and 1 was Hispanic. All completed two MRI visits and underwent surgical resection. The small sample and molecular-subtype eligibility requirement limit generalizability to older patients, unresectable or metastatic PDAC, and patients without adequate pretreatment biopsy tissue.
